# The Feasibility of Structural Health Monitoring Using the Fundamental Shear Horizontal Guided Wave in a Thin Aluminum Plate

**DOI:** 10.3390/ma10050551

**Published:** 2017-05-19

**Authors:** Jorge Franklin Mansur Rodrigues Filho, Nicolas Tremblay, Gláucio Soares da Fonseca, Pierre Belanger

**Affiliations:** 1 Department of Mechanical Engineering, Universidade Federal Fluminense, Niteróy 24220-900, Brazil; jorgemansur@id.uff.br (J.F.M.R.F.); glaucio@metal.eeimvr.uff.br (G.S.d.F.); 2 Department of Mechanical Engineering, École de Technologie Supérieure, Montreal, QC H3C 1K3, Canada; nicolas.tremblay.12@ens.etsmtl.ca

**Keywords:** structural health monitoring, ultrasonic guided waves, shear horizontal waves

## Abstract

Structural health monitoring (SHM) is emerging as an essential tool for constant monitoring of safety-critical engineering components. Ultrasonic guided waves stand out because of their ability to propagate over long distances and because they can offer good estimates of location, severity, and type of damage. The unique properties of the fundamental shear horizontal guided wave (SH_0_) mode have recently generated great interest among the SHM community. The aim of this paper is to demonstrate the feasibility of omnidirectional SH_0_ SHM in a thin aluminum plate using a three-transducer sparse array. Descriptions of the transducer, the finite element model, and the imaging algorithm are presented. The image localization maps show a good agreement between the simulations and experimental results. The SH_0_ SHM method proposed in this paper is shown to have a high resolution and to be able to locate defects within 5% of the true location. The short input signal as well the non-dispersive nature of SH_0_ leads to high resolution in the reconstructed images. The defect diameter estimated using the full width at half maximum was 10 mm or twice the size of the true diameter.

## 1. Introduction

Structural health monitoring (SHM) is rapidly emerging as an essential tool for continuous monitoring of safety-critical engineering components. In a typical SHM system, transducers are permanently installed so as to enable periodic assessment of the structure. From a comparison of features in the signals acquired at different times, damages can be detected. The recent development of the SHM system will soon become the cornerstone in changing from scheduled maintenance to condition-based maintenance. SHM can be performed using a vast array of methods, e.g., acoustic emission and fiber Bragg grating [1]. However, ultrasonic guided waves stand out because of their ability to propagate over long distances and because they can offer good estimates of location, severity, and type of damage [2,3].

Considerable efforts have been made on the use of the fundamental Lamb modes (A_0_ and S_0_) in SHM [4,5,6,7,8,9,10]. In order to generate the ultrasonic guided wave, SHM images the use of spatially distributed arrays [11,12] or phased arrays are required [13,14]. The images are then typically formed by a combination of a baseline subtraction approach and a delay-and-sum imaging algorithm [15]. More recently, several novel approaches to sparse array imaging have been proposed. For instance, minimum variance imaging was proposed to reduce the clutter in delay-and-sum images but requires a priori knowledge of the defects scattering patterns [16]. In correlation imaging [17], scattered fields from all possible defect locations are precomputed and stored in a dictionary. The precomputed scattered information is then compared with the actual wave field, and a correlation coefficient is calculated. These advanced approaches require knowledge of the scattering mechanism of the mode considered. On the other hand, the unique properties of the fundamental shear horizontal guided wave mode (SH_0_) have recently attracted great interest in the SHM community [18]. SH_0_ is the only non-dispersive ultrasonic guided wave mode; it is not affected by fluid loading and has good potential in detecting defects associated with composite material structures [19,20]. Moreover, SH_0_ will not convert to other guided wave modes when interacting with a defect perpendicular to the direction of propagation, therefore leading to increased sensitivity [21].

In order to ensure maximum monitoring coverage using a minimal number of transducers, omnidirectional transduction is preferred. Moreover, piezoelectric transducers are advantageous because they are lightweight, have a small footprint, and may be designed to survive harsh conditions. However, omnidirectional transduction of SH_0_ using piezoelectric material is difficult because a torsional surface stress is required. Two piezoelectric concepts have recently been proposed in the literature [22,23], which may be adapted to SH_0_ SHM. The non-dispersive nature of the SH_0_ mode combined with a broadband transducer may lead to a significant increase in the damage detection resolution because a very short input signal will be possible.

The aim of this paper is to demonstrate the feasibility of SH_0_ SHM on a thin aluminum plate representative of a simplified aerospace structure using a three-transducer sparse array. The first section of the paper describes the materials and methods including the details of the transducer used in this work, the finite element model, and the imaging algorithm. The second section presents a comparison of simulations and experiments as well as a discussion of the main findings. In the final section, conclusions are drawn.

## 2. Materials and Methods

### 2.1. Transducer

**Figure 1:**
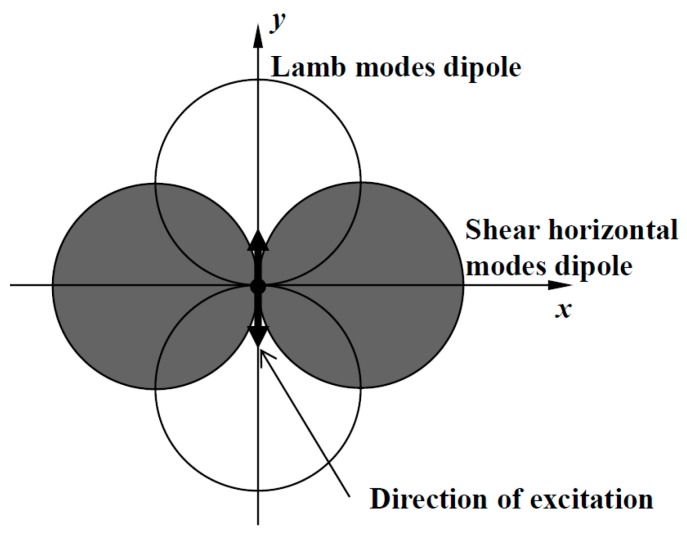
Pair of dipole patterns excited by a point surface shear source. The lobes of the Lamb modes dipole are in opposition of phase, whereas the lobes of the SH dipole are in phase.

The transducer used in this paper was adapted from Belanger and Boivin [22]. This transducer was shown to have an experimental SH_0_ mode selectivity of 17 dB and excellent omnidirectionality. Below the cutoff frequency thickness product of high order modes, a surface shear point source has a pair of dipoles as in Figure 1. The Lamb mode dipole is oriented in the direction of the excitation, and its lobes of the dipole are in opposition of phase. The SH dipole on the other hand is perpendicular to the direction of excitation and its lobes are in phase. Therefore, if an infinite number of surface shear point sources are superposed with the orientation of excitation varying between 0 and 360°, both Lamb modes would interfere destructively, whereas SH_0_ would interfere constructively. Hence, a torsional surface stress will generate pure SH_0_.

**Figure 2:**
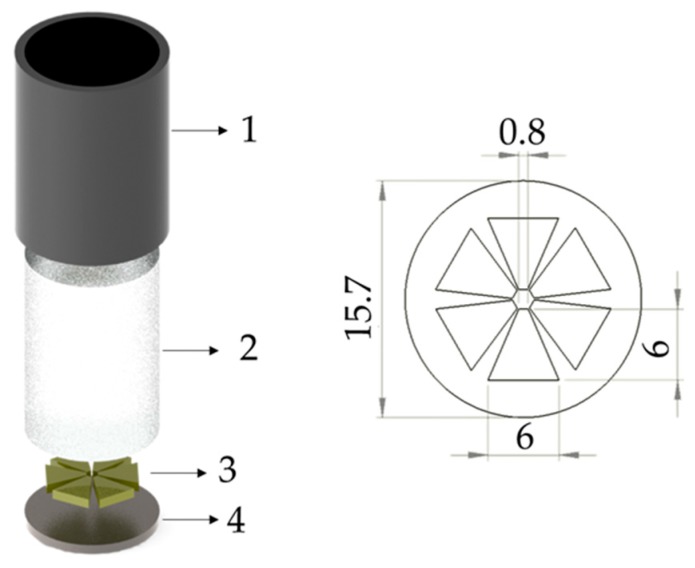
Schematic of the omnidirectional piezoelectric transducer assembly. The component description: 1—ABS plastic casing; 2—Epoxy–tungsten mix; 3—Piezoelectric PZT-5H patches; 4—Titanium plate.

The surface torsional stress is generated by six PZT-5H trapezoidal piezoelectric patches working in pure shear. In order to facilitate experimental development on multiple structures, transducer units including a matching layer, the piezoelectric elements, and a backing mass as shown in Figure 2 were assembled. The piezoelectric trapezoidal patches were bonded to a titanium plate using conductive silver loaded epoxy. The titanium plate was used for its intermediate shear acoustic impedance between PZT-5H and aluminum. An ABS plastic casing was used to protect the ceramics and to support the electrical connections. The case was filled with tungsten loaded epoxy. The transducer was designed to be broadband with a 150 kHz central frequency.

In the results section, the broadband nature of the transducer is demonstrated in a pitch-catch measurement using a pair of transducers.

### 2.2. Finite Element Model

**Figure 3:**
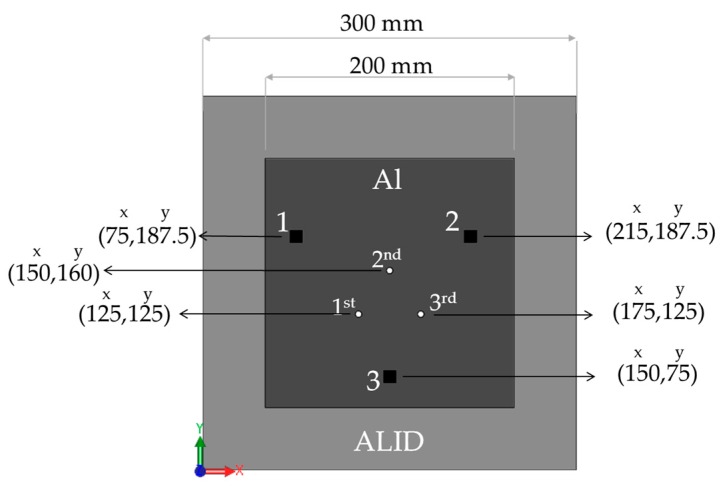
Schematic of the finite element plate. The dimensions of the absorbing boundaries and the locations of the transducers (black squares) and of the holes (white dots) are shown in millimeters. The wave field was simulated successively after adding each defect.

A finite element (FE) model was designed using the Abaqus Unified FEA (Version 6.13-4, Dassault Systemes, Providence, RI, USA) simulation package and was used to study the wave propagation and interaction with defects as well as to develop the imaging algorithm and validate the experimental results. A full 3D model using hexahedral elements sized at 15 elements per wavelength was used. Reflections from the plate edges were attenuated using absorbing boundaries with increasing damping [24]. The schematic presented in Figure 3 shows the plate dimensions and the location of the main features. A thin aluminum plate (*t* = 1.6 mm, *E* = 70.75 GPa, *ρ* = 2700 kg/m^3^ and *ν* = 0.337) was used to represent a simplified aerospace structure. A sparse array of three transducers with a location provided in Figure 3 was added to the plate. In the model, the torsional surface stress required for pure SH_0_ excitation was applied as a torsional displacement on the plate surface. The transducer itself was therefore not modeled. The same strategy was used in reception. An initial baseline of the wave propagation between each pair of transducers was simulated for a one cycle Hann windowed input signal centered at 150 kHz. The center frequency of the simulation was chosen based on the transducer presented in the previous section. Through thickness holes with a 5 mm diameter were successively added to Locations 1, 2, and 3 in the schematic of Figure 3. The wave field was simulated after adding each new hole.

### 2.3. Signal Processing

Baseline subtraction is a technique widely used in the SHM community [3,25,26,27]. In a baseline subtraction approach, the difference between signals acquired at two different times is used to provide information about the location and severity of defects that were introduced after the baseline and before the latest measurement. The advantage of this method is that it is possible to generate images of a defect location and severity using a small number of transducers. However, changes to the wave field due to causes other than defects such as temperature variation or different load scenario may lead to false positives.

**Figure 4:**
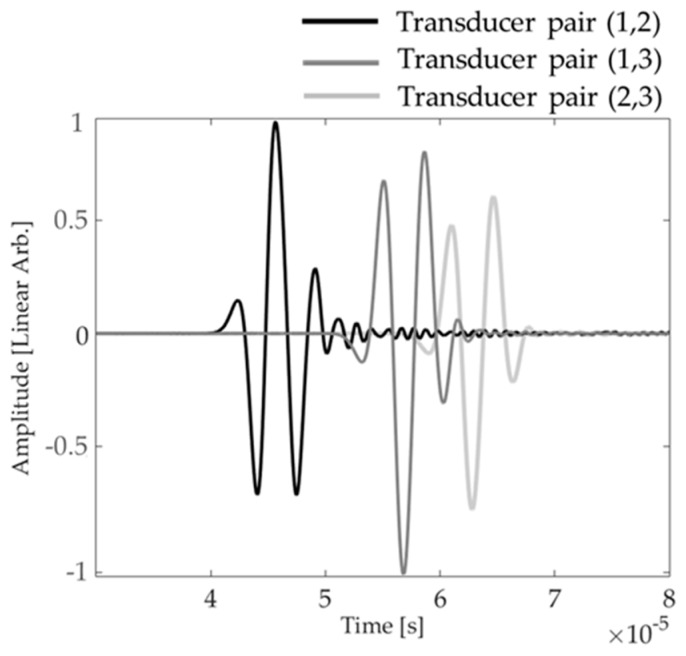
Example of subtracted signals containing a defect for all pairs of transducers.

In both simulations and experiments, an initial baseline was acquired for a plate in pristine conditions. A first defect was added and the wave field was saved. An image was formed to locate the defect and the first defect wave field then became the baseline for future measurements. In this work, the signals for each transducer pair (1,2), (1,3), and (2,3) were subtracted from their respective baselines, resulting in a subtracted signal for each pair. The subtracted signals were then normalized to the maximum value of any pair. Figure 4 shows an example of the subtracted signals in simulations for all pairs of transducers for the case with the 1st defect.

An imaging algorithm based on arrival time ellipses was used for defect localization [28,29]. The algorithm is based on the concept of triangulation. First, Equation (1) is used to calculate the time (t*_ij_*(*x*,*y*)) a wave with group velocity (V_gr_) would take to travel from the source position (*x_i_*,*y_i_*) to a specific point (*x*,*y*) on the plate and then to the receiver position (*x_j_*,*y_j_*). Mode selectivity of the transducer is important to ensure that a single mode is propagating. Single mode propagation leads to unambiguous images. As SH_0_ is non-dispersive, the group velocity is constant for all frequencies, and short input signals can be used. Short input signals lead to an improved imaging resolution.

(1) $$t_{ij}=\frac{\sqrt{{{(x}_{i}-x)}^{2}{+(y}_{i}{-y)}^{2}}+\sqrt{{{(x}_{j}-x)}^{2}{+(y}_{j}{-y)}^{2}}}{V_{\mathrm{gr}}}$$

**Figure 5:**
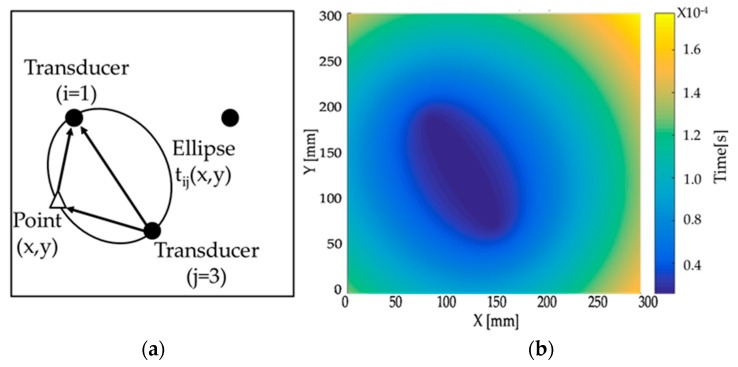
Schematic of the ellipse equation and the resulting map for all points in a 2D domain. (**a**) Equal travel times between a source and a sensor results in an ellipse; (**b**) Calculating the ellipse for all points in a domain leads to a map of travel time ellipses.

**Figure 6:**
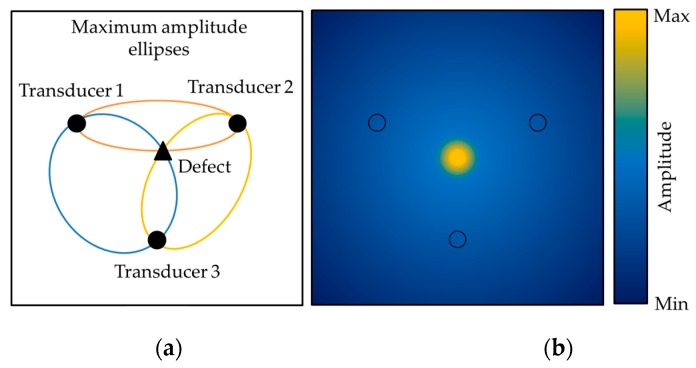
(**a**) Schematic of the effect caused by summing the ellipse generated by the baseline subtracted signals; (**b**) At a defect the ellipses are interfering constructively.

Figure 5 presents a schematic of the algorithm. The ellipse shows the points with equal travel time (t*_ij_*(*x*,*y*)). By scanning all points of the plate, a color scale maps as presented in Figure 5 can be obtained. Then, by comparing the baseline subtracted wave field with the travel time ellipses, it is possible to estimate the position of the defect. The defect location appears on an ellipse for a single pair of transducers. However, by using multiple pairs of transducers, the defect location can be estimated as the point at which all ellipses are crossing, therefore leading to constructive interference as shown in Figure 6 [29].

### 2.4. Experimental Setup

**Figure 7:**
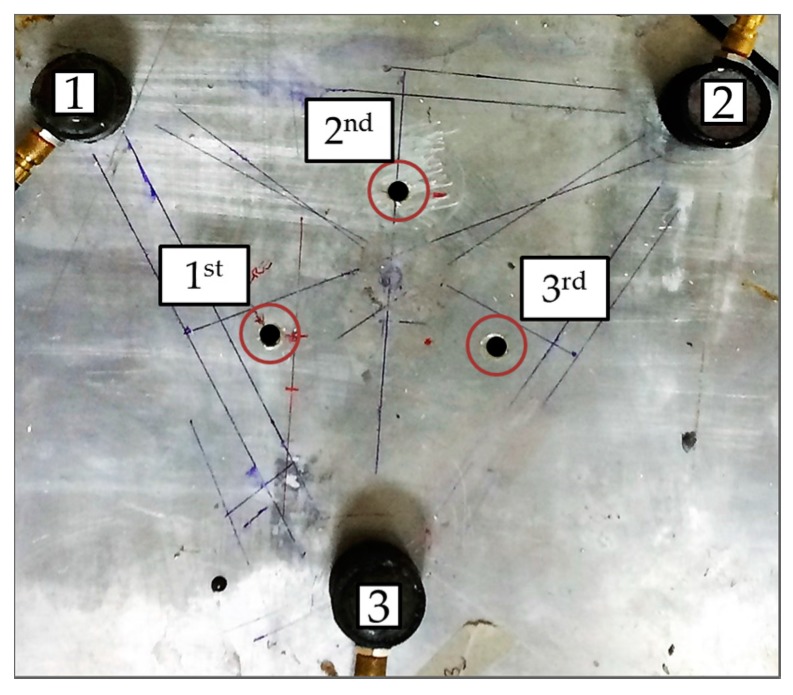
Experimental setup used in this paper. The omnidirectional SH_0_ transducers are identified with Numbers 1, 2, and 3. The through-thickness holes were drilled successively to ensure that the wave field for each condition was saved.

Figure 7 shows the experimental aluminum plate with three omnidirectional SH_0_ transducers. The transducers and defect locations are exactly the same as the simulations. The transducers were bonded to the plate using cyanoacrylate. A single cycle Hann windowed input signal centered at 150 kHz was generated using an Agilent arbitrary waveform generator (33521B, Agilent Technologies, Santa Clara, CA, USA) and was then amplified using a Ritec RPR-4000 pulser amplifier (Ritec, Warwick, RI, USA). An initial pristine condition baseline was acquired. A first through-thickness hole with a 5 mm diameter was drilled through the plate. After each drilling, the wave field was acquired and saved.

## 3. Results and Discussion

### 3.1. Transducer Time-Domain Analysis

**Figure 8:**
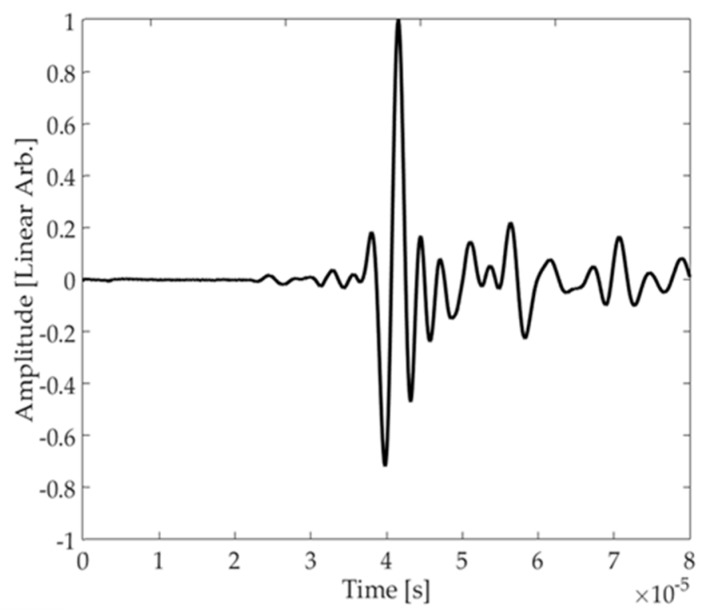
Pitch catch time-trace obtained from Transducer 1–3 in Figure 7.

Figure 8 presents the time trace obtained experimentally between Transducers 1 and 3 in Figure 7. Even if the one cycle Hann windowed toneburst is slightly distorted, the bandwidth of the transducer supports wide bandwidth signals. The signal arriving after SH_0_ contains A_0_ at an amplitude approximately 15 dB below SH_0_ as well as reflection from the edges of the plate. A_0_ is excited by this transducer because of the out-of-plane loading of the piezoelectric elements. Artifacts are expected from the small A_0_ component.

### 3.2. Comparison between FE and Experimental Results

**Figure 9:**
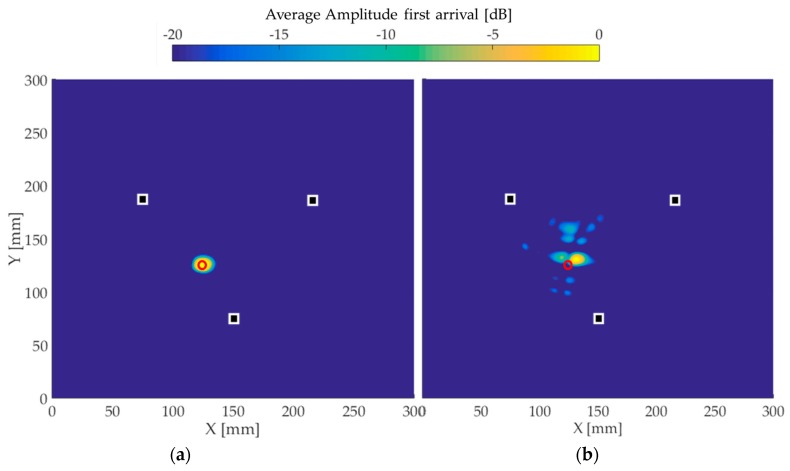
Reconstructed images for the first 5 mm through-thickness hole. The white squares represent the transducers positions and the red circles correspond to the defect position. (**a**) The image using simulated time traces and (**b**) the experimental image.

**Figure 10:**
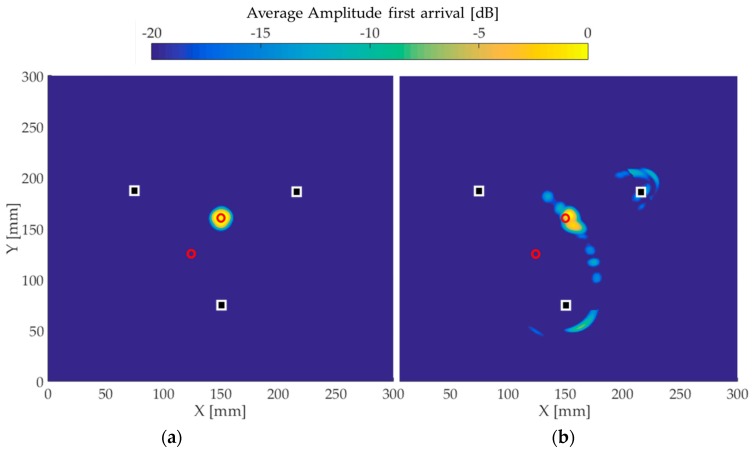
Reconstructed images for the second 5 mm through-thickness hole. The white squares represent the transducers positions and the red circles correspond to the defect position. (**a**) The image using simulated time traces and (**b**) the experimental image.

**Figure 11:**
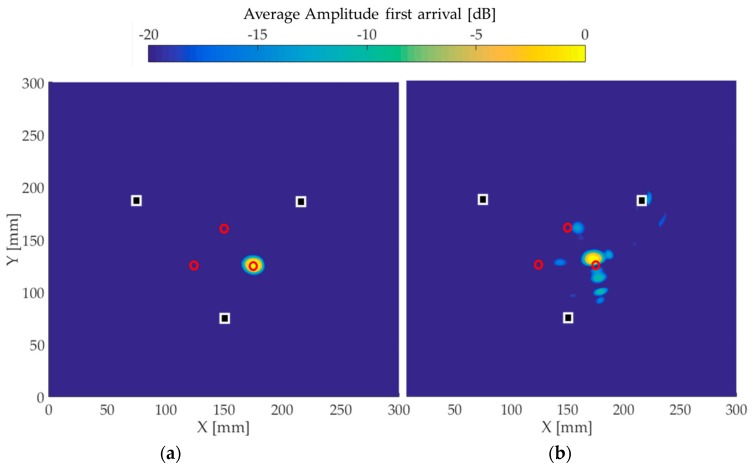
Reconstructed images for the third 5 mm through-thickness hole. The white squares represent the transducers positions and the red circles correspond to the defect position. (**a**) The image using simulated time traces and (**b**) the experimental image.

Figure 9, Figure 10 and Figure 11 present the image reconstructions of the wave fields obtained in (a) simulations and (b) experiments. The white squares represent the positions of the transducers, while the red circles correspond to the position of the defect. The amplitude scale is shown using a 20 dB threshold.

For the case with one defect, presented in Figure 9, the simulation image shows a peak amplitude in the exact position of the hole. However, in the experimental image, a small deviation of the defect position of approximately 5% is seen, and some reconstruction artifacts approximately 8 dB below the level of the amplitude generated by the defect are reconstructed.

Figure 10 shows the plots for the case when a second defect was added. In this case, the baseline was the wave field acquired to generate Figure 9. It is therefore expected that the first hole would not show on Figure 10 because it is already included in the baseline. Again, the simulation image shows a peak amplitude in the exact position of the hole. In experiments, the defect is still clearly visible with a small deviation in its position. Encouragingly, the image artifacts are of a level similar to those of the experimental image presented in Figure 9b.

Figure 11 presents the images when a third hole is drilled through the plate. The simulation is again in excellent agreement with the true position of the defect. The experimental image shows the same trend as those of Figure 9 and Figure 10. The amplitude level of the artifacts remains the same throughout all experimental images.

The simulation results show an excellent agreement with the true defect locations. Moreover, the amplitude in the images remains constant even after a third defect is added. This is very encouraging as this information can be used to assess the severity of the defects. Experimental deviations in terms of defect location occurred, but the error remains under 5%. The slight error in the defect locations are thought to be due to the slight anisotropy of the cold rolled plate leading to small variations in the SH_0_ velocity as a function of the direction of propagation. As in simulations, the amplitude of the defect in the experimental images appears to be constant for up to three identical defects.

The artifacts in the simulated images was always below −20 dB. However, the experimental images contained imaging artifacts in the region of 8 dB below the amplitude of the defect. However, the amplitude level of the artifacts remained constant for up to three identical defects. The experimental artifacts are likely due to the transducers slightly exciting A_0_. Due to assembling difficulty, the transducers also all have slightly different performance.

When compared with results shown in the literature using longer input signals, typically in the region of 5 cycles with A_0_ or S_0_ [29,30], the advantage of using a broadband SH_0_ transducer becomes clear. The experimental and simulated full width at half maximum of the defects is 10 mm in all cases or approximately twice the true size of the defect.

## 4. Conclusions

This paper demonstrated the feasibility of an SH_0_ sparse array SHM system on a thin aluminum plate. Finite element simulations have shown that a three omnidirectional SH_0_ transducer array can be used to accurately locate through-thickness holes using the baseline subtraction approach and triangulation of the signals acquired by each pair of transducers. Moreover, the non-dispersive nature of SH_0_ combined with a broadband transducer enabled the use of a very short input signal. This resulted in high-resolution images and accurate defect localization. The experimental validation was conducted on a 1.6 mm aluminum plate. Although the experimental defect locations were not as precise as the simulations, the results were in excellent agreement. The defect diameter estimated using the full width at half maximum was 10 mm or twice the size of the true diameter. Future work will focus on validating the method for other types of defects and verify the temperature stability.
